# Machine learning methods for protein-protein binding affinity prediction in protein design

**DOI:** 10.3389/fbinf.2022.1065703

**Published:** 2022-12-16

**Authors:** Zhongliang Guo, Rui Yamaguchi

**Affiliations:** ^1^ Division of Cancer Systems Biology, Aichi Cancer Center Research Institute, Nagoya, Aichi, Japan; ^2^ Division of Cancer Informatics, Nagoya University Graduate School of Medicine, Nagoya, Aichi, Japan

**Keywords:** machine learning, deep neural network, protein-protein interaction, binding affinity, protein design

## Abstract

Protein-protein interactions govern a wide range of biological activity. A proper estimation of the protein-protein binding affinity is vital to design proteins with high specificity and binding affinity toward a target protein, which has a variety of applications including antibody design in immunotherapy, enzyme engineering for reaction optimization, and construction of biosensors. However, experimental and theoretical modelling methods are time-consuming, hinder the exploration of the entire protein space, and deter the identification of optimal proteins that meet the requirements of practical applications. In recent years, the rapid development in machine learning methods for protein-protein binding affinity prediction has revealed the potential of a paradigm shift in protein design. Here, we review the prediction methods and associated datasets and discuss the requirements and construction methods of binding affinity prediction models for protein design.

## 1 Introduction

Protein-protein interactions play a central role in biological activities, including signal transduction, cell metabolism, and immune system (Osaki et al., 2004; De Las Rivas and Fontanillo, 2010; Guo, 2014; Szeto et al., 2020). Determining the protein-protein interactions helps researchers elucidate biological phenomena, find causes of diseases, and design new drugs (Ryan and Matthews, 2005; Carter, 2006; Fleishman et al., 2011; Sliwkowski and Mellman, 2013; Guo et al., 2014; Rosell and Fernandez-Recio, 2018). As a representative example, cancer immunotherapy has been proven to be highly effective against certain cancer types and has attracted considerable attention (Varela-Rohena et al., 2008; Restifo et al., 2012). In immunotherapy, T cells recognize and attack cancer cells by binding the complementary determining regions (CDRs) of the T cell receptors (TCRs) to the peptide presented by a major histocompatibility complex (pMHC) on cancer cell (Smith-Garvin et al., 2009). The interactions between TCRs and the target cancer antigen must be correctly evaluated to design TCRs that efficiently recognize cancer cells.

The equilibrium dissociation constant (K_d_) or Gibbs free energy (ΔG), which can be derived from the K_d_, is commonly used to quantify protein-protein interactions (Wang et al., 2004; Kastritis et al., 2011; Moal and Fernandez-Recio, 2012; Jankauskaitė et al., 2019). As K_d_ or inhibition constant (K_i_) measurements of protein complexes are sometimes performed simultaneously with X-ray crystallography experiments, some datasets contain protein-protein binding affinity data together with the 3D structures (Wang et al., 2004; Kastritis et al., 2011; Borrman et al., 2017). These datasets can therefore be used for the prediction of binding affinity based on the 3D structures. However, the experimental measurement procedure for K_d_ is labor-intensive and time-consuming, which sometimes requires sophisticated experimental equipment (Zhou et al., 2016).

As one of the most important application fields of protein design, the protein-protein interaction data of antibody-antigen binding or TCR-pMHC recognition provide crucial information for immunotherapy, which can characterize the amino acid sequences and structures of antibody or TCR binding to a target. Recently, single-cell sequencing has been used in immune profiling to generate high-throughput quantitative data on the interaction of TCRs or BCRs (B cell receptors) and antigens (Bentzen et al., 2016). Using the samples collected from donors, the sequence of TCR or BCR in each cell is identified *via* single-cell sequencing, and the binding strength of the receptor and antigen is measured based on the counts of dextramer that carry multiple antigens and can be quantified based on the feature barcode (10x Genomics, 2020). Models trained on the single cell immune profiling dataset are validated on conventional TCR-antigen binding datasets (Sidhom et al., 2021). Although single cell immune profiling can measure thousands of T cells simultaneously, the size of TCR sequence space is estimated to exceed 10^20^ (Zarnitsyna et al., 2013), making it impossibly difficult to find the optimal TCR using samples obtained from donors or those that are randomly generated.

To circumvent the above-mentioned limitations of experimental measurement, methods for predicting binding affinity using molecular dynamics simulations, empirical energy functions, and machine learning methods have been developed (Chothia and Janin, 1975; Horton and Lewis, 1992; Jiang et al., 2002; Ma et al., 2002; Zhang et al., 2005; Audie and Scarlata, 2007; Su et al., 2009; Flower et al., 2010; Panday and Alexov, 2022). Molecular dynamics simulations provide highly accurate predictions at the cost of high computational intensity (de Vries et al., 2010; De Paris et al., 2015). Empirical energy functions are used in protein-ligand and protein-protein affinity prediction. Although this method is less computation-demanding, further improvements are necessary to achieve the accuracy required for molecule design (Kastritis and Bonvin, 2010). Compared with the previous two methods, machine learning methods have been developed to handle complex tasks that do not work well with manually-curated functions, such as natural language processing and computer vision (LeCun et al., 2015; Goodfellow et al., 2016). In recent years, machine learning has demonstrated its potential in various fields of natural science, including physics, chemistry, and biology (Stanev et al., 2018; Wu et al., 2019; Jumper et al., 2021; Li M. et al., 2022). In the field of structural biology, AlphaFold and RosettaFold have shown high accuracy in predicting protein structures based on amino acid sequences (Baek et al., 2021; Jumper et al., 2021).

In the past decade, numerous machine learning methods on the prediction of protein-protein interactions have been published, including classification models to identify pairs of proteins forming complex, protein-protein interaction site predictions, and binding affinity predictions (Casadio et al., 2022; Hu et al., 2022; Li S. et al., 2022). In particular, the high-throughput feature of machine learning models is highly valuable for virtual screening and protein design.

**FIGURE 1:**
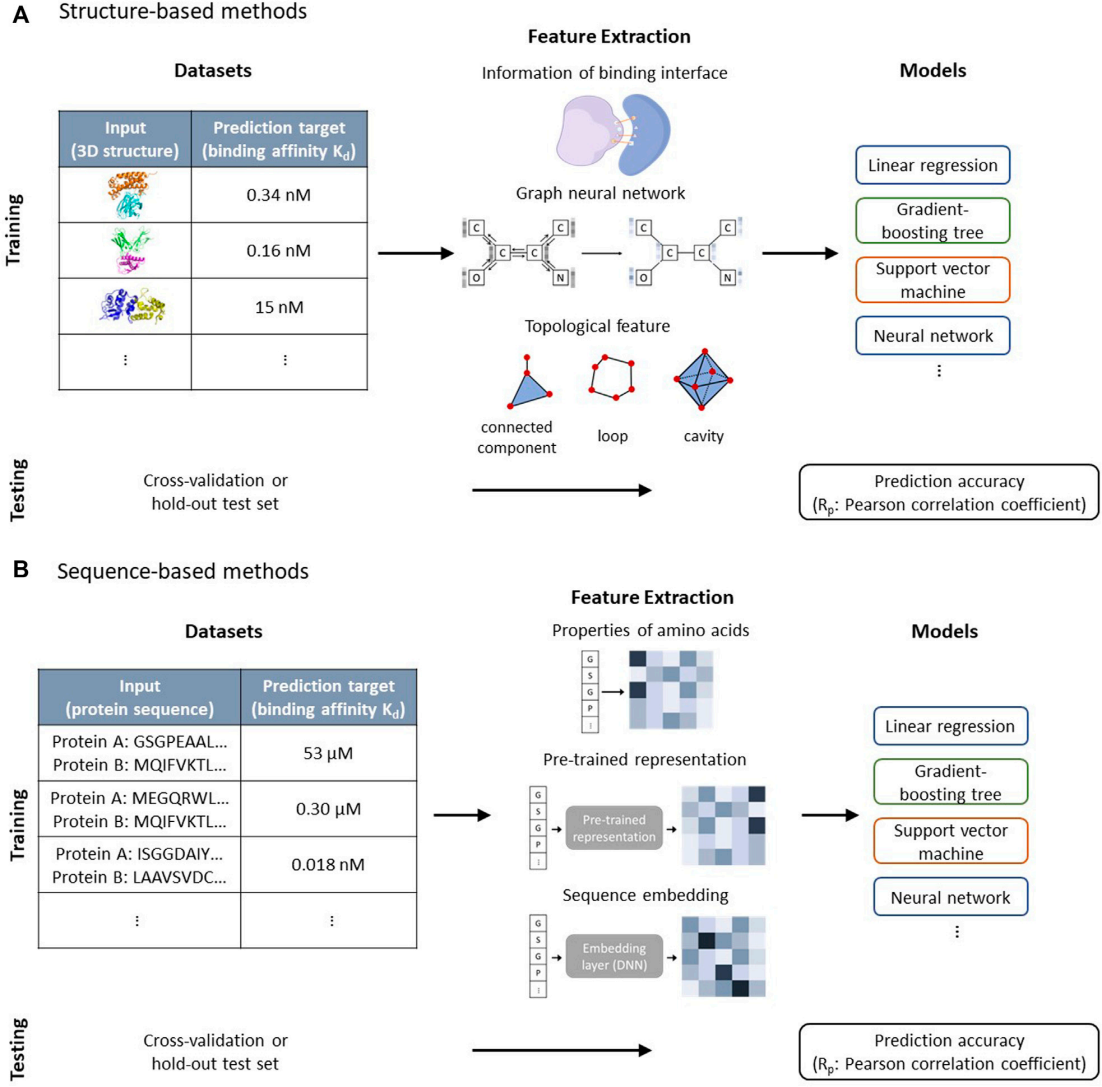
**(A)** Structure-based methods use the 3D structure of proteins as input to predict the binding affinities. The information related to the binding surface, graph neural network, and topology features are used as descriptors and combined with machine learning models to predict binding affinities. Cross-validation and hold-out test set are used to validate prediction accuracies of models. **(B)** Sequence-based methods use protein sequences to predict protein-protein binding affinities. The properties of amino acids, pre-trained representation, and sequence embedding layer that is trained with the prediction model are used to extract features of protein sequences, which act as inputs for machine learning models. Cross-validation and hold-out test set are used to validate prediction accuracies of models.

In this perspective, we focus on quantitative prediction models for protein-protein binding affinity and associated datasets, which can be further deployed in protein design. Figure 1 shows how machine learning models utilize resources in datasets to predict the binding affinity and different featurization methods. The general case in protein design is to engineer a protein so that its property falls within a predetermined range. Considering that the subsequent experiments are time-consuming, it is extremely important in practical applications to correctly estimate the property to reduce false positives and false negatives. General reviews on the application of machine learning for protein-protein interaction are given by Casadio et al. (2022), Hu et al. (2022), and Li S. et al. (2022). We discuss issues in prediction models of protein-protein binding affinity with regard to their application in protein design, for which highly accurate quantitative prediction is required. We also provide guidance on how to take advantage of information from different datasets to construct accurate prediction models and discuss the usage of the recently developed highly accurate protein structure prediction model in data augmentation.

## 2 Datasets

Machine learning models are designed based on the type of data they will be used on. For datasets that are extracted from the Protein Data Bank (PDB) (wwPDB consortium, 2019), both the 3D structures and amino acid sequences can be used as the inputs, and K_d_, K_i_, or IC_50_ (the concentration at 50% inhibition) will be the prediction target (Li et al., 2014; Liu et al., 2015; Liu et al., 2017). Recently, single-cell RNA-sequencing has been applied to determine the immune profiles in order to find TCRs or BCRs that bind specifically to a certain target (10x Genomics, 2020). For the sequencing datasets, machine learning models take the nucleic acid or amino acid sequences of proteins as the input and predict their binding specificity (Abbasi et al., 2020; Fischer et al., 2020). In this section, we briefly discuss the data type, size, and other aspects that may affect the prediction accuracy of machine learning models (Table 1).

**TABLE 1:** Datasets of protein-protein binding affinities used in machine learning predictions.

Dataset	Data size	Data type	Binding affinity	Experimental conditions	Available source
PDBbind v2020 Wang et al. (2004)	2,852	complex structures (PDB)	K_d_, K_i_ or IC_50_	—	http://www.pdbbind.org.cn/index.php
Structure-based benchmark for protein-protein binding affinity Kastritis et al. (2011)	144	complex structures (PDB) and unbound component structures (PDB)	K_d_, ΔG	method, temperature, pH	Supporting information
SKEMPI 1.1 Moal and Fernandez-Recio (2012)	3,047 (158 PDB structures)	complex structures (PDB of wild type), mutations	K_d_, ΔG	method, temperature	https://life.bsc.es/pid/mutation_database/
SKEMPI 2.0 Jankauskaitė et al. (2019)	7,085 (345 PDB structures)	complex structures (PDB of wild type), mutations	K_d_, ΔG	method, temperature	https://life.bsc.es/pid/skempi2/
ATLAS Borrman et al. (2017)	694 (123 PDB structures)	complex structures (PDB or template PDB)	K_d_, ΔG	method, temperature	http://atlas.wenglab.org/web/index.php
10x single cell immune profiling dataset 10x Genomics (2020)	136,477 cells (55,221 distinct TCR clonotypes)	TCR sequence	UMI counts	—	https://www.10xgenomics.com/resources/datasets

### 2.1 PDBbind

The PDBbind dataset is a collection of complex structures extracted from PDB with binding affinities determined experimentally (Wang et al., 2004). The first version of PDBbind dataset was published in 2004 and only contained protein-ligand binding data. Since the update in 2008, protein-protein complexes, protein-nucleic acid complexes, and nucleic acid-ligand complexes have also been included in the dataset (Li et al., 2014; Liu et al., 2015; Liu et al., 2017). Until 2020, the PDBbind dataset was updated annually. In the first week of each year, new protein structure data deposited in PDB during the previous year were included in the dataset. A program was designed to determine if the PDB files contain protein complex structure data or not and to classify the complex structure file into one of the four classes (protein-ligand complexes, protein-protein complexes, protein-nucleic acid complexes, and nucleic acid-ligand complex) (Liu et al., 2015). Another program was used to screen the primary reference of the complex structure file. Articles containing binding affinity data were manually curated and the PDB IDs, binding affinities, and comments were recorded in the dataset. In the current release (PDBbind 2020), there are a total of 23,496 entries in the dataset, comprising 19,433 protein-ligand complexes, 2,852 protein-protein complexes, 1,052 protein-nucleic acid complexes, and 149 nucleic acid-ligand complexes. For building a binding affinity prediction model of protein-protein complexes, the structure and amino acid sequence data of the complexes can be obtained from the PDB files and K_d_, K_i_, or IC_50_ values recorded in the dataset are the prediction targets.

### 2.2 Structure-based benchmark for protein-protein binding affinity

The structure-based benchmark for protein-protein binding affinity is an assembled dataset of 144 protein-protein complexes (Kastritis et al., 2011). The dataset is composed of three complex classes. Class A contains antigen-antibody complexes (19 cases), class E is the enzyme-containing class (61 cases), and class O (“other”) includes complexes that do not fall into the previous two classes. For each of the complexes, both K_d_ and ΔG are reported in the dataset along with the pH, temperature, and experimental methods used. As the protein-protein interaction can induce a conformation change, both the structures of complexes and unbound components are available in this dataset. Compared with other datasets, the structure-based benchmark for protein-protein binding affinity is a small dataset, but the included additional information of pH and temperature are very valuable for improving the prediction accuracy. In addition, the structure data of unbound components can be used to construct models that predict the binding affinity of two proteins from different structure resources. This is the most common case in practice.

### 2.3 SKEMPI and SKEMPI 2.0

SKEMPI (Structural database of Kinetics and Energetics of Mutant Protein Interactions) is a database describing the changes of binding affinities and other kinetics characteristics upon mutations (Moal and Fernandez-Recio, 2012). SKEMPI 2.0 is an updated version of the SKEMPI database (Jankauskaitė et al., 2019). In SKEMPI 1.1, there were 158 PDB entries and binding affinity data for 3,047 mutants. In SKEMPI 2.0, the numbers of PDB entries and mutants have increased to 345 and 7,085, respectively. A large part of the data in SKEMPI was collected from the structure-based benchmark for protein-protein binding affinity (Kastritis et al., 2011), ASEdb (Thorn and Bogan, 2001), PINT (Kumar and Gromiha, 2006), and the associated references. The additional data included in SKEMPI 2.0 were collected from datasets associated with published literature and from the references of the updated version of protein–protein docking and binding affinity benchmarks (Vreven et al., 2015). The changes in binding energy upon mutations, as well as information on the experimental methods and temperatures, were acquired manually from the associated literature and added to the database. For the structure data, the PDB IDs of the wild-type proteins were recorded. In instances, where the crystal structures of the mutants were also reported in the papers, the structure data were also included in the dataset.

### 2.4 ATLAS

The ATLAS (Altered TCR Ligand Affinities and Structures) database is a collection of the binding affinity of a TCR to a pMHC(Borrman et al., 2017). The database includes a total of 694 entries with 123 PDB structures. Each entry includes the TCR, peptide, MHC, binding affinity, and PDB ID if the crystal structure is known. For the complexes whose crystal structure is not reported, a template PDB ID for the complex is included for further calculation of the structure based on the template. ATLAS contains information similar to SKEMPI and SKEMPI 2.0, but the dataset is focused on TCR-pMHC complexes. As the design of proteins with the desired properties often involves introducing mutations to proteins from a specific family, this dataset’s focus on a certain domain is well suited for constructing efficient prediction models for protein design based on the same domain. Other datasets, such as the AB-Bind (Sirin et al., 2016), antibody-antigen docking and affinity benchmark (Guest et al., 2021), focus on the antibody-antigen interaction and are therefore useful for antibody design.

### 2.5 10x single cell immune profiling dataset

Unlike the previously described datasets, the 10x single cell immune profile dataset is generated *via* single-cell RNA-sequencing (10x Genomics, 2020). T cells obtained from four healthy donors were labeled with antibodies to find CD8^+^ T cells with the ability to kill cancer or virus-infected cells. The dataset comprises data of 136,477 cells, which includes 55,221 distinct TCR clonotypes. The specificities of the TCRs with regard to binding to pMHCs were identified using dextramers that carry antigens. For this, 44 dextramer reagents with six negative controls were mixed with the cells and the binding strength was quantified using the counts of unique molecular identifier (UMI) on the dextramer. Although the data obtained using this method are noisy, this method represents a high-throughput method for identifying TCRs with high binding strength to a target pMHC.

## 3 Machine learning methods

Machine learning is a research field that focuses on the data to find patterns, build models for prediction or explanation, and understand the relationship underlying the data (Bishop, 2006; Mitchell, 2013). Machine learning methods, from simplest linear regression to deep learning (Seal, 1967; Cortes and Vapnik, 1995; Tin Kam Ho, 1995; Breiman, 1996; Friedman, 2002; LeCun et al., 2015; Goodfellow et al., 2016), have been developed for decades and are implemented in science, finance, healthcare, and other fields (Dixon et al., 2020; Guo et al., 2020; Varoquaux and Cheplygina, 2022; Zhang et al., 2022). In structural biology, machine learning methods have been used to predict the structure of proteins based on their amino acid sequences, design new molecules for enzyme inhibition, and predict the protein-protein interactions (Vamathevan et al., 2019; Baek et al., 2021; Jumper et al., 2021; Romero-Molina et al., 2022). In this section, we focus on regression models for protein-protein interaction prediction (Table 2). In protein design, it is common to restrict the binding strength to a particular range; thus, the prediction models should be able to assess the binding affinity accurately.

**TABLE 2:** Machine learning methods for protein-protein binding affinity prediction.

Model	Data type	Features	Model details	Accuracy
Vangone and Bonvin Vangone and Bonvin (2015)	3D structure	network of inter-residue contacts and non-interacting surface	linear regression	R_p_ = 0.73 on a benchmark of 79 protein-protein complexes
mmCSM-PPI Rodrigues et al. (2021)	3D structure	graph-based signatures and complementary features	extra trees	R_p_ = 0.75 on SKEMPI 2.0
GeoPPI Liu et al. (2021)	3D structure	graph neural network	gradient-boosting tree	R_p_ = 0.58 on SKEMPI, R_p_ = 0.52 on SKEMPI 2.0
TopNetTree Wang et al. (2020)	3D structure	persistent homology, CNN	gradient-boosting tree	R_p_ = 0.85 on SKEMPI, R_p_ = 0.79 on SKEMPI 2.0
PerSpect-EL Wee and Xia (2022)	3D structure	persistent homology, physical properties	ensemble model (CNN + GBT)	R_p_ = 0.853 on SKEMPI
PPI-Affinity Romero-Molina et al. (2022)	3D structure	ProtDCal	support vector machine	R_p_ = 0.77 on SKEMPI
PPA-Pred Yugandhar and Gromiha (2014)	protein sequence	amino acid properties from AAindex and other resources	multiple regression	R_p_ = 0.909^a^ on 135 complexes selected from the structure-based benchmark
ISLAND Abbasi et al. (2020)	protein sequence	kernel representation	support vector regression	R_p_ = 0.44 on structure-based benchmark
PIPR Chen et al. (2019)	protein sequence	pre-trained embedding representation	residual recurrent convolutional neural network	R_p_ = 0.873 on SKEMPI
PIPR + S2F Xue et al. (2022)	protein sequence	pre-trained sequence embedding	residual recurrent convolutional neural network	R_p_ = 0.264 on a subset of SKEMPI 2.0
TcellMatch Fischer et al. (2020)	TCR sequence	sequence embedding	neural network	R_p_ = 0.63 on 10x dataset

^a^ The average of correlations obtained by nine prediction models for nine subclasses in the dataset.

### 3.1 Structure-based methods

Structure-based methods use the 3D structure of a protein as model input to predict the binding affinity. As the data contain extensive information regarding the protein-protein interface, the feature vector is well-designed to capture the essential information for each model. Vangone and Bonvin used the network of inter-residue contacts and the non-interacting surface as the descriptors (Vangone and Bonvin, 2015). The performance of a linear regression model was tested on a benchmark dataset of 79 protein-protein complexes; the Pearson correlation coefficient (R_p_) of the experimental ΔΔG (binding affinity change caused by mutations) and predicted ΔΔG was 0.73. Rodrigues et al. proposed a method to predict binding affinity based on graph-based signatures, which described the distance patterns between atoms on the binding interface (Rodrigues et al., 2021). Complementary features, including experimental conditions and non-covalent contacts, were also a part of these models. An extra trees model, trained on the graph-based signatures and complementary features, was shown to have best performance on the SKEMPI 2.0 dataset with R_p_ = 0.75.

Liu et al. proposed a machine learning model combining a graph neural network (GNN) with a gradient-boosting tree (GBT) (Liu et al., 2021). The GNN used the message passing architecture (Gilmer et al., 2017) to generate the feature vector of the complex, and a self-supervised training scheme was used to train the GNN (Doersch and Zisserman, 2017). Perturbations were applied to the coordinates of the protein side chain, the GNN was used to encode the perturbated 3D structure to a hidden vector, and the model was trained to reduce the discrepancy between the reconstructed coordinate and the original coordinate. This self-supervised training procedure was considered to help the GNN capture important information on the interactions between the proteins in the complex. The performance of GBT using the descriptor generated by the GNN was R_p_ = 0.58 and 0.52 on SKEMPI and SKEMPI 2.0 datasets, respectively.

Wang et al. developed a topology-based network to capture the geometric and topological pattern of the complex efficiently (Wang et al., 2020). The features were calculated using persistent homology (Edelsbrunner et al., 2002; Zomorodian and Carlsson, 2005), which was also applied in material science and protein-ligand binding affinity prediction and has been known as a powerful tool in machine learning (Kovacev-Nikolic et al., 2016; Shirai and Nakamura, 2019). The features generated by persistent homology were processed using a convolutional neural network (CNN) to extract high-level feature vectors. Combining the feature vectors with information of atom types, the final input vectors were created and GBT was used to predict the ΔΔG. The model was named TopNetTree and achieved an R_p_ of 0.85 and 0.79 on SKEMPI and SKEMPI 2.0 datasets, respectively.

Another topology-based model named PerSpect-EL was proposed by Wee and Xia who combined persistent homology with ensemble learning to improve the prediction accuracy (Wee and Xia, 2022). CNN models were trained to predict binding affinity from the persistent homology features, and a GBT model was trained using the physical properties of the protein complex for binding affinity prediction. Meta learners conducted the final prediction based on the CNN outputs and GBT output. The ensemble model achieved an R_p_ of 0.853 on SKEMPI dataset.

PPI-Affinity is a web tool that predicts the binding affinity using support vector machine and other classic machine learning models (Romero-Molina et al., 2022). Accepting thousands of features generated by ProtDCal as input (Romero-Molina et al., 2019), the model showed a performance of R_p_ = 0.77 on SKEMPI dataset. As the ProtDCal is a general-purpose program for generating 3D-structure descriptors, it is said that some machine learning models can extract binding information from the universal descriptors to predict the binding affinity.

### 3.2 Sequence-based methods

Sequence-based methods take the amino acid sequence as the input and directly predict the binding affinity. The featurization methods include substitution matrix representation (SMR), position-specific scoring matrix (PSSM), and other embedding methods developed for natural language processing (Zvelebil and Baum, 2008; Dubitzky et al., 2013; Yang et al., 2018). Yugandhar and Gromiha proposed PPA-Pred (Yugandhar and Gromiha, 2014), which was a multiple regression model using amino acid properties from AAindex and other resources as features (Kawashima and Kanehisa, 2000; Ofran and Rost, 2007). A dataset with 135 complexes selected from the structure-based benchmark for protein-protein binding affinity were divided to nine subclasses. One model was built for each subclass and the correlations ranged from 0.739 to 0.992. ISLAND (In SiLico protein AffiNity preDictor) combined a kernel representation of protein sequences with the support vector regression to predict the binding affinity (Abbasi et al., 2020). The R_p_ of the measured and predicted ΔG was 0.44 on the structure-based benchmark for protein-protein binding affinity. Chen et al. developed an end-to-end model to predict the binding affinity from the amino acid sequence based on a recurrent convolutional neural network (RCNN) (Chen et al., 2019). Compared with other models using autocovariance or composition-transition-distribution descriptors as features, a Siamese residual RCNN with a pretrained embedding representation of protein sequences provided the best performance (R_p_ = 0.873). Another model based on pre-trained embedding and residual RCNN was proposed by Xue et al. (Xue et al., 2022). This method was different from other sequence-based methods, as the structure information and functions of proteins were used in the pre-training stage to generate sequence embeddings containing structural and functional information of the proteins. The performance of the model (R_p_ = 0.264) was reported using a homology and structure similarity-base data splitting method on a subset of SKEMPI 2.0. Fischer et al. considered the UMI counts in 10x single cell immune profiling dataset as the binding strength of TCR-pMHC complex (Fischer et al., 2020). A model named TcellMatch was developed to predict the pMHC count based on the TCR sequence, surface protein counts, and other covariates (donor, total count of mRNA, and negative-control pMHC counts). The *R* ^2^ of the prediction was 0.63 on the 10x dataset.

## 4 Discussion

In this study, we introduced several datasets and models for binding affinity prediction. In this section, we will discuss issues regarding the practical application in protein design. As machine learning methods offer high-throughput prediction with high accuracy, they are desirable tools for screening newly designed proteins for specific binding to a target. Recent reviews on this topic cover prediction models of protein-protein interaction that are mainly classification models (Casadio et al., 2022; Hu et al., 2022; Li S. et al., 2022); however, there has been little discussion on the quantitative prediction of machine learning models. Since it is an essential prerequisite in protein design to correctly estimate whether the properties of the engineered protein are within the desired range, the current situation and problems of the quantitative prediction for protein-protein binding affinity should be clarified for further improvement.

The most pressing problem is that there is no widely accepted evaluation method for protein-protein binding affinity models. As shown in Table 2, the accuracies of the models discussed in Section 3 are tested on different datasets. In addition, different studies use different data splitting methods in cross-validation to report prediction accuracies on the same dataset. Among them, ten-fold cross-validation is used in most of the models. However, multiple studies have reported that the accuracies can be overestimated due to the similarity of data in the training dataset and test dataset (Park and Marcotte, 2012; Hamp and Rost, 2015; Abbasi and Minhas, 2016; Liu et al., 2021). Liu et al. (2021) and Xue et al. (2022) evaluated the models using devised data splitting methods based on homology and structure similarity. As each model is designed to exhibit high performance when evaluated on a specific dataset with a specific data split, it is nearly impossible to compare the prediction accuracy fairly without a common evaluation method. A desirable dataset for evaluation is expected to be large, having different types of protein-protein interactions, such as antibody-antigen, enzyme-substrate, and other complexes. In addition, both low- and high-binding affinity complexes are necessary to evaluate the performances of models for varying types of data. Moreover, as different models use different information as inputs, experimental conditions and other auxiliary information should be recorded with the sequential and structural information of the complex. However, as building a large and comprehensive dataset is time-consuming and can be a future solution for model evaluation, a practical evaluation method using currently available dataset is testing models on SKEMPI 2.0 dataset, which combines binding affinity data from multiple sources and has gained a lot of attention due to its wide use in recent research. To identify the issues in performance evaluation due to the similarity of data between the training and test datasets, both ten-fold cross-validation and similarity-based data splitting should be applied to evaluate the accuracies of models, as the deviation between different data splitting methods shows the robustness of the model when trained using different training data, which is an important aspect in the model evaluation.

Since the task of binding affinity prediction for protein design is different from general protein-protein binding affinity prediction, in which the prediction targets are in the same protein family, such as antibody, TCR, or enzyme, the evaluation method for prediction models used in protein design should be specified accordingly. As an example, in antibody or TCR design, mutations are introduced to a specific protein to enhance the binding affinity to a particular range (Makowski et al., 2022). Therefore, an evaluation method is required to evaluate the prediction ability of models in a subspace that covers the mutants generated in protein design.

While this model evaluation is relatively simple, models can perform poorly in the high binding affinity region. This is because the binding affinities of most antibodies or TCRs are in the low affinity range, and training data for the model may not be sufficient to learn the pattern of high-affinity proteins. This type of problem also exists in other fields, such as materials design (Lookman et al., 2019), where one of the solutions is active learning combined with simulations and experiments. As an example, binding affinities of proteins generated by a Bayesian optimization method can be calculated using highly accurate simulation models. The data are collected and used to improve the prediction accuracy of machine learning models.

In addition to simulations and experiments, recently developed highly accurate structure prediction models are also important tools in the study of protein-protein interactions (Baek et al., 2021; Jumper et al., 2021). As the structure of the designed protein or the complex is rarely known, the structures predicted by machine learning models are used as input for structure-based binding affinity prediction models (Bryant et al., 2022). Combining the binding affinity dataset with the virtual structure database generated by AlphaFold is expected to alleviate some of the data shortage in protein-protein binding affinity prediction (Szklarczyk et al., 2021; Varadi et al., 2022). Other advances in experimental measurement methods, such as cryo-electron microscopy, can also promote data accumulation in this research area (Yip et al., 2020).

In order to improve the prediction accuracy, various techniques have been used in the models. Rodrigues et al. included the experimental conditions and other auxiliary information in the model (Rodrigues et al., 2021). As temperature and pH have been shown to change binding affinities (Kastritis and Bonvin, 2010; Dias and Kolaczkowski, 2017), using this additional information offers a simple yet effective approach to increase the prediction accuracy. Moreover, protein complex data without binding affinity information have been used to train the feature extraction model (Liu et al., 2021). For sequence-based models, the structure information can be used to pre-train the embeddings of protein sequences (Xue et al., 2022). The properties of amino acids and feature vectors generated as general-purpose descriptors also have shown predictive ability in various studies (Yugandhar and Gromiha, 2014; Romero-Molina et al., 2022). To integrate different models using varying features, ensemble learning is extremely helpful to combine outputs of models to obtain better performance (Wee and Xia, 2022).

Although machine learning methods have been extensively used in various fields of science to address pertinent issues, it has some limitations as well. One of the common problems is the lack of data, which is also present in the protein-protein binding affinity prediction. However, the rapid development of related research fields raises expectations for the emergence of large datasets that can enable highly accurate prediction models. Experimental methods such as cryo-electron microscopy increase the accumulation of structure data, prediction models for protein structures are used to generate virtual protein structures, and simulation methods can predict the binding affinities based on protein structures. Therefore, a high-performance model trained on a large dataset is expected to appear in the near future and make itself an indispensable tool for protein design, which can be further applied to various fields associated with healthcare, material sciences, and energy.

## Data Availability

The original contributions presented in the study are included in the article/Supplementary Material, further inquiries can be directed to the corresponding author.
